# Implementation and validation of the extended Hill-type muscle model with robust routing capabilities in LS-DYNA for active human body models

**DOI:** 10.1186/s12938-017-0399-7

**Published:** 2017-09-02

**Authors:** Christian Kleinbach, Oleksandr Martynenko, Janik Promies, Daniel F. B. Haeufle, Jörg Fehr, Syn Schmitt

**Affiliations:** 1 Institute for Engineering and Computational Mechanics, University of Stuttgart, Pfaffenwaldring 9, 70569 Stuttgart, Germany; 2 Biomechanics and Biorobotics, Stuttgart Research Centre for Simulation Sciences (SRC SimTech), University of Stuttgart, Allmandring 28, 70569 Stuttgart, Germany; 3 Multi-Level Modeling in Motor Control and Rehabilitation Robotics, Hertie Institute for Clinical Brain Research, University of Tübingen, Otfried-Müller-Strasse 25, 72076 Tübingen, Germany

**Keywords:** Hill-type muscle model, LS-DYNA, Muscle routing, Human body model, Finite element analysis, Biomechanics

## Abstract

**Background:**

In the state of the art finite element AHBMs for car crash analysis in the LS-DYNA software material named *MAT_MUSCLE (*MAT_156) is used for active muscles modeling. It has three elements in parallel configuration, which has several major drawbacks: restraint approximation of the physical reality, complicated parameterization and absence of the integrated activation dynamics. This study presents implementation of the extended four element Hill-type muscle model with serial damping and eccentric force–velocity relation including $Ca^{2+}$ dependent activation dynamics and internal method for physiological muscle routing.

**Results:**

Proposed model was implemented into the general-purpose finite element (FE) simulation software LSDYNA as a user material for truss elements. This material model is verified and validated with three different sets of mammalian experimental data, taken from the literature. It is compared to the *MAT_MUSCLE (*MAT_156) Hill-type muscle model already existing in LS-DYNA, which is currently used in finite element human body models (HBMs). An application example with an arm model extracted from the FE ViVA OpenHBM is given, taking into account physiological muscle paths.

**Conclusion:**

The simulation results show better material model accuracy, calculation robustness and improved muscle routing capability compared to *MAT_156. The FORTRAN source code for the user material subroutine dyn21.f and the muscle parameters for all simulations, conducted in the study, are given at https://zenodo.org/record/826209 under an open source license. This enables a quick application of the proposed material model in LS-DYNA, especially in active human body models (AHBMs) for applications in automotive safety.

## Background

It is hard to imagine that the development of modern vehicles can be done without wide utilization of computer aided engineering (CAE), including state-of-the-art numerical simulations software. These tools not only offer opportunities to the car manufacturers for saving time and costs during the design phase, but also to model and predict future product lifecycle. One of the most demanding and regulated domains is vehicle safety and therefore crash simulations. For more than a quarter of a century, complete vehicles are modelled virtually as finite element models with all significant details, including material and geometrical properties. The same approach was then applied to the human body, and in the last decade several detailed finite element human body models (HBMs) were presented [1–3]. Joint simulations with a combined application of car and human body models allow for prediction of in-crash behaviour and possible injuries for occupants or pedestrians with a sufficient accuracy throughout all stages in development, replacing expensive crash tests using car prototypes and Anthropomorphic Test Devices (ATDs).

These so-called virtual testing methods will gain importance in the future, as the current trends of active safety systems and autonomous vehicles become available on the market. Active safety systems, in contrast to traditional passive safety systems, react preventively prior to a crash to avoid or mitigate a possible impact. This requires active HBMs (AHBMs) that are able to reproduce human behaviour in normal driving situations, as well as the behaviour in the in-crash phase. The same requirements exist for the second trend of autonomous vehicles, where generic driving or sitting positions no longer exist, but where occupants can move freely. From these requirements, three challenges for AHBM modelling arise. The first being the implementation of active muscles as mathematical models of the muscle-tendon complex (MTC) including the activation dynamics, which will be addressed in the contribution. The second challenge is to model biologically relevant neural controllers to enable accurate forward dynamics (FD) simulations of human reactions and voluntary motion in all kind of traffic scenarios. The third challenge is the choice of parameters for AHBMs, as only a correct representation of both the passive components and active components will result in an accurate representation of a living human. Most current HBMs have a passive stiffness which is too high, see e.g. [4, 5]. On the one hand, this is to compensate for the active components still missing. On the other hand, this is because the source for the parameters are almost exclusively post mortem human subjects, where tissue modulus and no-load strain differ from living tissue depending on the postmortem time and post-mortem rigor [6].

**Fig. 1:**
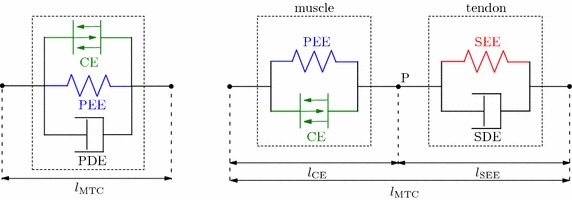
Schematic structures of the Hill-type muscle models. With* CE* contractile element,* PEE *parallel elastic element,* PDE* parallel damping element,* SEE* serial elastic element,* SDE* serial damping element.** a** Structure of the LSDYNA muscle model *MAT 156.** b** Structure of the implemented four element muscle model

In the state of the art finite element AHBMs [7, 8] for car crash analysis the LS-DYNA material named *MAT_MUSCLE (*MAT_156) is used for modeling active muscles. This material is an advanced version of the previous model *MAT_SPRING_MUSCLE [9] for discrete elements, that is no longer being supported. *MAT_156 represents a Hill-type muscle model which consists of three parallel elements: contractile element (CE), parallel elastic element (PEE) and a parallel damping element (PDE), see also Fig. 1a. The implementation was done by Dr. J. A. Weiss based on prior studies and reviews on different Hill-type model element configurations by [10–12]. The implemented configuration was chosen due to its simplicity, ease of parameters derivation from the experiments and computational efficiency. However, in the publication [12] it was pointed out, that an element configuration with better approximation of the physical reality should be used in simulations if possible. Such an extended Hill-type muscle model should have a clear separation between muscle fibres and tendon structures. For a correct representation of the MTC dynamics an additional internal degree of freedom is required to decouple active muscle fibre and elastic tendon dynamics. Subsequent studies investigating the role of the serial elastic element have shown, that such simplifications and assumptions can lead to instabilities produced by force-velocity or force-length relation formulations [13], incorrect energy storage and release in the interaction with the environment [14, 15], unrealistic high-frequency oscillations [16] and differences in muscle force magnitude [17]. All these effects, mentioned in publications above, directly influence the explicit integration scheme used in LS-DYNA thus impacting speed, accuracy and robustness of simulations with AHBMs.

Usually, muscles and tendons wrap around bones or joints in both steady state conditions and while performing movements, consequently a physiological muscle path representation (muscle routing) is essential for FD simulations [18, 19]. Slight changes in the muscle line of action will lead to inaccurate muscle forces and resulting moments due to incorrect lever arms and muscle length. To model physiological muscle paths in finite element HBMs different muscle routing methods can be used. Fixed lever arms or the via-point method [20, 21] are the most simple options and the usage of contact detection [22] would be the most sophisticated method. According to [23] a via-point method should be preferred for *MAT_156 using the *ELEMENT_BEAM_PULLEY keyword in LS-DYNA. However, it is unclear if this method is applicable, as there exist so far no successful implementation of this method in AHBMs to the authors knowledge.

Additional disadvantages result from the way parameters are set for *MAT_156 in LS-DYNA. For a number of parameters predefined curves are required, e.g. muscle activation level vs. time or stress vs. the stretch ratio. These curves need to be defined beforehand or might be calculated during the runtime through the *DEFINE_CURVE_FUNCTION keyword and PIDCTL [24] options. This approach is limited, cumbersome and error prone. Instead, muscle parameters and constants found in anatomico-physiological literature should be used directly. Also, some disadvantages exist for muscle activation dynamics. Predefined muscle activation level vs. time curves cannot represent the activation dynamics correctly and to model the dynamics more precisely the activation level has to depend on the muscle length. The complete activation dynamics can be included efficiently in the material model for the muscle itself.

This study addresses the problems mentioned above and presents an implementation of the extended Hill-type muscle model with serial damping and eccentric force-velocity relation proposed by Haeufle et al. [25] into LS-DYNA code. The muscle model is additionally extended by activation dynamics and a method for physiologial muscle routing. The complete description of the model, its implementation, verification and validation are given in the next sections.

## Methods

In this section the complete model, its implementation in LS-DYNA, and the verification and validation set-up are described.

### Muscle model

One of today’s most popular and widely used macroscopic muscle model was proposed by Hill in 1938 [26] on the basis of experiments with frog muscles. The most important feature is a direct relation between muscle force and contraction velocity. Furthermore the model is also referred to as a first order muscle model, which means, that the muscle elements have neither mass nor inertia, and only an axial force is applied on the skeletal model between origin and insertion point of the muscle. During the past years some disadvantages of the original Hill model were found and there have been many publications with the aim of further developing and improving this model.

In the publication of Haeufle et al. [25], a modified Hill-type muscle model was proposed with improved serial damping and eccentric force-velocity relation. This model consists of four simple mechanical elements: an active contractile element (CE), which is controlled by the activation level *q*; parallel (PEE) and serial (SEE) nonlinear spring elements and a serial damping element (SDE). The model’s structure was based on a previous study by Günther et al. [16] which determined, that the model with a force-dependent SDE provides the best results in a comparison with constant parallel, constant serial, and force-dependent parallel damping elements. The structure of the MTC of the Haeufle model is shown in Fig. 1b, with two clearly separated parts modeling the active muscle fibres (CE + PEE) and the passive tendon and aponeurosis structures (SEE + SDE). The main equations of the muscle model are presented in the following. They were taken from [16, 19, 25, 27], where more detailed explanations can be found if needed. Furthermore, a comprehensive study on the influence of individual parts and their model formulation is given in [28].

As shown in Fig. 1b the muscle model features an internal degree of freedom which is described by $l_\text {CE}$ . The lengths of the passive elements are equal to

1 $$\begin{aligned} l_\text {PEE}&= l_\text {CE} \end{aligned}$$

2 $$\begin{aligned} \text {and}~l_\text {SDE}&=l_\text {SEE}\,. \end{aligned}$$

Then the total MTC length is

3 $$\begin{aligned} l_\text {MTC} = l_\text {CE} + l_\text {SEE}\,. \end{aligned}$$

The force equilibrium at point P between the muscle fibre and the tendon part is described in [16] as:

4 $$\begin{aligned} F_\text {PEE}(l_\text {CE})\,+\,F_\text {CE}(l_\text {CE},\dot{l}_\text {CE},q) = F_\text {SEE}(l_\text {MTC},l_\text {CE}) + F_\text {SDE}(\dot{l}_\text {MTC},\dot{l}_\text {CE},l_\text {CE},q)\,. \end{aligned}$$

#### Contractile element (CE)

The contractile element represents the active fibre bundles of the MTC. The force of the contractile element $F_\text {CE}$ is therefore dependent on the muscle activity *q*, the contraction velocity $\dot{l}_\text {CE}$ as well as the length-dependent isometric force $F_\text {isom}(l_\text {CE})$. It is expressed by the equation

5 $$\begin{aligned} F_\text {CE}(l_\text {CE}, \dot{l}_\text {CE}, q) = F_\text {max} \left( \frac{qF_\text {isom}+A_\text {rel}}{1-\frac{\dot{l}_\text {CE}}{B_\text {rel} l_\text {CE,opt}}}-A_\text {rel}\right) \,. \end{aligned}$$

The factors $A_\text {rel}$ and $B_\text {rel}$ are so-called normalized Hill parameters, where $A_\text {rel}$ is normalized with the maximum isometric force $F_\text {max}$ and $B_\text {rel}$ with the optimal fibre length $l_\text {CE,opt}$ [16,  p. 64]. The subscript ‘rel’, for relative, indicates the normalization. The optimal muscle fibre length at which the isometric force reaches the maximum value is $l_\text {CE,opt}$ . The isometric force $F_\text {isom}$ depends on the length of the contractile element and is calculated as follows:

6 $$\begin{aligned} F_\text {isom}(l_\text {CE}) = \exp {\left( -\left| \frac{\frac{l_\text {CE}}{l_\text {CE,opt}}-1}{\Delta W_\text {limb}} \right| ^{\nu _\text {CE,limb}}\right) }\,. \end{aligned}$$

This equation represents the bell-shaped force-length relationship of the CE element. The width of the normalized bell curve $\Delta W_\text {limb}$ and the exponent $\nu _\text {CE,limb}$ may be chosen differently for the ascending and descending limb of the force-length curve.

When calculating the Hill parameters, it is distinguished between an eccentric $\dot{l}_\text {CE} > 0$ (lengthening fibres) and a concentric case $\dot{l}_\text {CE} \le 0$ (shortening fibres). Please note, that in physiological muscle experiments, where shortening work of muscle fibres is examined, the sign convetion for the contraction velocity is usually the opposite (shortening fibres have a positive velocity) to ensure that the work of shortening muscles is positive. In the concentric case, the Hill parameters are:

7 $$\begin{aligned} A_\text {rel}(l_\text {CE},q)=A_\text {rel,0} \cdot L_{\text {A}_\text {rel}}(l_\text {CE}) \cdot Q_{\text {A}_\text {rel}}(q)\,, \end{aligned}$$

8 $$\begin{aligned} B_\text {rel}(l_\text {CE},q)=B_\text {rel,0} \cdot L_{\text {B}_\text {rel}}(l_\text {CE}) \cdot Q_{\text {B}_\text {rel}}(q)\,. \end{aligned}$$

The auxiliary variables for the calculation of the Hill parameters are divided into length- and activation-dependent components. The length-dependent parameters are defined as:

9 $$\begin{aligned} L_{\text {A}_\text {rel}}=\left\{\begin{array}{cl} 1, & \quad l_\text {CE}< l_\text {CE,opt}\\ F_\text {isom}, & \quad l_\text {CE} \ge l_\text {CE,opt}\end{array}\right. , L_{\text {B}_\text {rel}}(l_\text {CE})=1 \end{aligned}$$

and the activation-dependent as:

10 $$\begin{aligned} Q_{\text {A}_\text {rel}}(q)=\frac{1}{4}(1+3q)\,, \end{aligned}$$

11 $$\begin{aligned} Q_{\text {B}_\text {rel}}(q)=\frac{1}{7}(3+4q)\,. \end{aligned}$$

The equations in the eccentric case can be derived from Eq. (5) as mentioned in [25] and thus they are formulated as:

12 $$\begin{aligned} A_\text {rel,e} = - F_e \cdot q \cdot F_\text {isom} \end{aligned}$$

and

13 $$\begin{aligned} B_\text {rel,e} = \frac{B_\text {rel}(1-F_e)}{S_e \left( 1+ \frac{A_\text {rel}}{qF_\text {isom}} \right) }\,. \end{aligned}$$

#### Parallel elastic element (PEE)

The PEE represents passive properties of the muscle fiber and the collagenous connective tissue surrounding the muscle belly. As soon as the length of the contractile element exceeds the resting length of the parallel elastic element $l_\text {PEE,0}$, it also contributes to the force developed by the MTC. Mathematically this is expressed as:

14 $$\begin{aligned} F_\text {PEE}=\left\{ \begin{array}{cl} 0\, & \quad l_\text {CE}< l_\text {PEE,0} \\ K_\text {PEE}(l_\text {CE,opt}-l_\text {PEE,0})^{\nu _\text {PEE}}\,. &{} \quad l_\text {CE} \ge l_\text {PEE,0}\end{array}\right. \end{aligned}$$

The spring stiffness $K_\text {PEE}$ is influenced by the optimal fibre length, the width of the bell curve and the maximum isometric force. It is calculated by:

15 $$\begin{aligned} K_\text {PEE}=\mathcal {F}_\text {PEE}\frac{F_\text {max}}{(l_\text {CE,opt}(\Delta W_\text {desc}+1-\mathcal {L}_\text {PEE,0}))^{\nu _\text {PEE}}}\,. \end{aligned}$$

The resting length is defined as $l_\text {PEE,0}=\mathcal {L}_\text {PEE,0} \cdot l_\text {CE,opt}$, hence $\mathcal {L}_\text {PEE,0}$ is the resting length normalized by $l_\text {CE,opt}$ , $\Delta W_\text {desc}$ is width of $F_\text {isom}(l_\text {CE})$ on a descending limb.

#### Serial elastic element (SEE)

Since structures similar to muscle tissue are also present in the tendon, their elastic properties are similar. The serial elastic element has a nonlinear or linear spring behaviour depending on the deflection $l_\text {SEE}$. When $l_\text {SEE}<l_\text {SEE,0}$ the tendon is relaxed and does not generate any force. In the range of $l_\text {SEE,0}<l_\text {SEE}<l_\text {SEE,nll}$ it has a nonlinear characteristic, and a linear characteristic for $l_\text {SEE} \ge l_\text {SEE,nll}$:

16 $$\begin{aligned} F_\text {SEE}(l_\text {SEE})=\left\{ \begin{array}{ll} 0, & \quad l_\text {SEE} <l_\text {SEE,0}\\ K_\text {SEE,nl}(l_\text {SEE}-l_\text {SEE,0})^{\nu _\text {SEE}}\,, &{} \quad l_\text {SEE}<l_\text {SEE,nll}\\ \Delta F_\text {SEE,0}+K_\text {SEE,l}\cdot (l_\text {SEE}-l_\text {SEE,nll})\,. &{} \quad l_\text {SEE} \ge l_\text {SEE,nll}\end{array}\right. \end{aligned}$$

The length $l_\text {SEE,nll}$ of the SEE at the transition from nonlinear to linear characteristic, the exponent $\nu _\text {SEE}$, and the nonlinear and linear stiffness factors $K_\text {SEE,nl}$ and $K_\text {SEE,l}$ are defined by the following formulas:

$$\begin{aligned} l_\text {SEE,nll}&= (1+\Delta U_\text {SEE,nll})\cdot l_\text {SEE,0}\,, \\ \nu _\text {SEE}&= \Delta U_\text {SEE,nll}/\Delta U_\text {SEE,l}\,, \\ K_\text {SEE,nl}&= \Delta F_\text {SEE,0}/(\Delta U_\text {SEE,nll}l_\text {SEE,0})^{\nu _\text {SEE}}\,, \\ K_\text {SEE,l}&= \Delta F_\text {SEE,0}/(\Delta U_\text {SEE,l}l_\text {SEE,0})\,. \end{aligned}$$

The complete description of these independent parameters can be found in [16, Fig. 4, p. 69].

#### Serial damping element (SDE)

The force-dependent serial damping element reduces unphysiological high-frequency oscillations in the tendon part of the muscle model. As a side effect this also increases numerical efficiency [16]. The force-dependent damping of the material damping element is calculated as:

17 $$\begin{aligned} F_\text {SDE}(l_\text {CE},\dot{l}_\text {SDE},q)= d_\text {SDE,max} \cdot \left( (1{-}R_\text {SDE}) {\cdot } \frac{F_\text {CE}{+}F_\text {PEE}}{F_\text {max}}{+}R_\text {SDE} \right) \dot{l}_\text {SDE}\,, \end{aligned}$$

with the maximum absorption value of

18 $$\begin{aligned} d_\text {SDE,max}=D_\text {SDE}\frac{F_\text {max}A_\text {rel,0}}{l_\text {CE,opt}B_\text {rel,0}}\,, \end{aligned}$$

using the dimensionless scaling factor $D_\text {SDE}$ and minimum damping value $R_\text {SDE}$ .

#### Contraction dynamics

Inserting Eq. (5) into Eq. (4) for the force equilibrium yields a quadratic equation of the form:

19 $$\begin{aligned} 0=C_2 \cdot \dot{l}_\text {CE}^2+C_1 \cdot \dot{l}_\text {CE}+C_0\,. \end{aligned}$$

This equation must be solved for the contraction velocity $\dot{l}_\text {CE}$ at each time step. Subsequent integration gives the solution for the internal muscle model degree of freedom—the length of the contractile element $l_\text {CE}$. Since the coefficients $C_1$ and $C_0$ are always less than zero for our configuration, the solution for the contraction dynamics is given as:

20 $$\begin{aligned} \dot{l}_\text {CE}=\left\{ \begin{array}{cl} \frac{-C_1-\sqrt{C_1^2-4 \cdot C_2 \cdot C_0}}{2 \cdot C_2}\,, &{} \quad \dot{l}_\text {CE} \le 0\\ \frac{-C_{1,\text {e}}+\sqrt{C_{1,\text {e}}^2-4 \cdot C_{2,\text {e}} \cdot C_{0,\text {e}}}}{2 \cdot C_{2,\text {e}}}\,. &{} \quad \dot{l}_\text {CE} > 0\end{array}\right. \end{aligned}$$

In this equation, the index *e* denotes that eccentric Hill parameters must be computed from Eqs. (12, 13). The coefficients $C_0$, $C_1$ and $C_2$ are determined as follows:

$$\begin{aligned} C_2&=d_\text {SDE,max}\left( R_\text {SDE}-\left( A_\text {rel}-\frac{F_\text {PEE}}{F_\text {max}}\right) \left( 1-R_\text {SDE}\right) \right) \,,\\ C_1&=-C_2 \cdot \dot{l}_\text {MTC}-D_0-F_\text {SEE}+F_\text {PEE}-F_\text {max}A_\text {rel}\,,\\ C_0&=D_0 \cdot l_\text {MTC}+l_\text {CE,opt} \cdot B_\text {rel}\left( F_\text {SEE}-F_\text {PEE}-F_\text {max} q F_\text {isom}\right) \,, \end{aligned}$$

with the additional coefficient

21 $$\begin{aligned} D_0=l_\text {CE,opt} \cdot B_\text {rel} \cdot d_\text {SDE,max}\left( R_\text {SDE}+\left( 1-R_\text {SDE}\right) \left( qF_\text {isom}+\frac{F_\text {PEE}}{F_\text {max}}\right) \right) \,. \end{aligned}$$

#### Activation dynamics

In the application of muscle models, not only the muscle dynamics itself, but also muscle activation dynamics needs to be considered. Activation dynamics is the link between stimulation input from the nervous system and the activity level of a muscle. For the proposed muscle model, two different muscle activation strategies are implemented: one depending only on the neural activation level (STIM) by Zajac [11] and another, which takes into account length-dependent sensitivity of $Ca^{2+}$ level change by Hatze [29]. These two activation dynamics are outlined below.

The first implemented activation dynamics by Zajac [11] was extended by [16] by adding a minimum muscle activity level $q_0$ to represent the fact that in reality a muscle is never physiologically completely inactive ($q \ne 0$). The differential equation for the activation dynamics therefore is noted as:

22 $$\begin{aligned} \dot{q} = \frac{1}{\tau _\text {act}}\left( STIM - STIM\left( 1-\beta _\text {q}\right) \left( q-q_0\right) - \beta _\text {q}\left( q-q_0\right) \right) \,. \end{aligned}$$

In this equation *STIM* is the input. It is the neural stimulation that emanates from the nervous system and varies from 0 to 1. The output is *q*, the CE element activation level with a possible range of $q_0 \le q \le 1$. It represents the concentration of free $Ca^{2+}$ ion in the muscle. $\tau _\text {act}$ is the activity time constant and $\beta _\text {q}$ is the ratio between time constant on activation and deactivation. Thus, for $\beta _\text {q} > 1$ the deactivation time constant is less than that of the activation.

The second activation dynamics implemented is a two-step approach introduced by Hatze [29]. In this approach, the activity level *q* depends on both the length of the contractile element $l_\text {CE}$ and the free $Ca^{2+}$ ion concentration. The activity level is calculated as follows:

23 $$\begin{aligned} q = \frac{q_0+(\rho \cdot \gamma _\text {rel})^3}{1+(\rho \cdot \gamma _\text {rel})^3}\,. \end{aligned}$$

The $Ca^{2+}$ ion concentration is accounted for in the differential equation as $\gamma _\text {rel}$:

24 $$\begin{aligned} \dot{\gamma }_\text {rel} = m(STIM-\gamma _\text {rel}) \text {, with } \gamma _\text {rel}(t_0)=0\,, \end{aligned}$$

and the relative CE length is included in $\rho$:

25 $$\begin{aligned} \rho = c \cdot \eta \frac{(k-1)}{\left( k-l_\text {CE,rel}\right) }l_\text {CE,rel}\,. \end{aligned}$$

Here *m*, *c* and $\eta$ are constants and $l_\text {CE,rel}$ is the ratio between the contractile element length $l_\text {CE}$ and the optimal fibre length $l_\text {CE,opt}$. Thus the length-dependent $Ca^{2+}$ ion sensitivity is taken into account, namely the relation that the longer the contractile element the higher the $Ca^{2+}$ sensitivity. In other words, stretched muscles produce a larger force at the same stimulation level compared to an already contracted muscle [30]. In addition, the $Ca^{2+}$ sensitivity contributes to low-frequency stiffness of the muscle, which is defined as the change in the equilibrium muscle force relative to a change in the equilibrium length with constant stimulation [31].

#### Muscle length offset and muscle routing

To enable physiological muscle path representation for the extended Hill-type muscle model several routing methods could be considered. In advanced modelling frameworks the muscle path is usually redirected either by specific points, so called via-points, or by surfaces of geometrical objects (e.g. in OpenSim [32]). See [33] additionally for an in-depth review and comparison of routing methods in biomechanical models. It was decided to use the via-point approach as described in [20], because it is possible to implement this method with the standard routing elements available for seatbelts in LS-DYNA. This method has proven to be reliable, as it is used in almost every crash simulation involving occupant models. To implement this, it is necessary to divide the MTC into muscle element and seatbelt elements, as only the latter can be routed. Therefore, an offset in length $l_\text {offset}$ is introduced, defined as the difference between the actual length of the muscle beam element length $l_\text {beam,mus}$ in the model and the length of the entire MTC $l_\text {MTC}$:

26 $$\begin{aligned} l_\text {MTC}&= l_\text {beam,mus} + l_\text {offset}\\&= l_\text {beam,mus} + l_\text {offset,1} + l_\text {offset,2}\,.\nonumber \end{aligned}$$

**Fig. 2:**
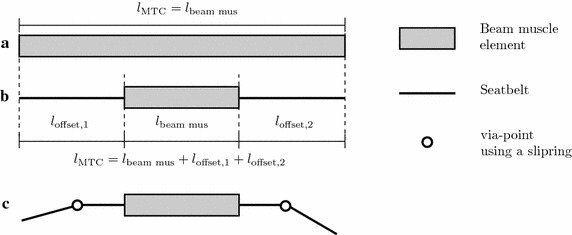
Comparison of the length relations for the muscle routing approach with the via-point method.** a** A full beam element with Hill-type muscle material, where the beam element represents the entire MTC.** b** A shortened beam element with Hill-type muscle material extended by seatbelt elements, and** c** the via-point routing method with two via-points. For the latter two, the muscle force is still calculated based on the entire MTC length (muscle + seatbelt), however, it is acting only in the beam element. This approach allows to use the slipring routing method of LS-DYNA

If necessary an offset can be added on both ends of the muscle beam element, to allow for two or more via-points, see Fig. 2. Standard seatbelt elements can be attached to the end of the muscle beam element and all the standard routing methods of LS-DYNA e.g. sliprings can be used. The seatbelt elements can move through a slipring node freely, while at the same time, the muscle model internally works with the correct length and dynamics of the entire MTC. To preserve the muscle dynamics it is required, that the stiffness of the seatbelt elements is orders of magnitude higher compared to the stiffness of the muscle elements. For the example in "Application in the ViVA OpenHBM Arm with routing" a stiffness of $1\times 10^6$ N/m was used for the seatbelt elements.

### LS-DYNA implementation

It is possible to include self-written code in the LS-DYNA FE solver through so-called ‘User Subroutines’. These subroutines have to be written in FORTRAN and can, among other options, be used to define user materials [24]. The muscle model described in "Muscle model" was implemented in LS-DYNA as a user material for truss elements to simulate the active contraction behaviour, as well as the passive spring and damping effects of human muscles. The FORTRAN code is available at https://zenodo.org/record/826209.

**Fig. 3:**
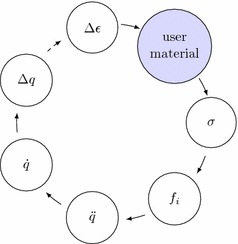
Implementation of the user material subroutine into LS-DYNA workflow

The explicit integration scheme in LS-DYNA, shown in Fig. 3, is updating the element strain $\Delta \epsilon$ in each timestep based on the nodal displacement $\Delta u$. Material models translate the strain $\Delta \epsilon$ to stress $\sigma$, which yield nodal forces $f_i$. These forces result in nodal acceleration $\ddot{u}$, which are integrated to nodal velocity $\dot{u}$ and displacement $\Delta u$ for the next timestep.

It should be pointed out, that material subroutines require an element stress as a return value. In the concept of the muscle model, only forces are calculated. Since truss elements can only have axial stress, the stress was calculated from the muscle force and the element cross-section area via

27 $$\begin{aligned} \sigma = \frac{F_\text {MTC}}{A}\,. \end{aligned}$$

If the material card for user-defined material models is specified in an input deck, LS-DYNA internally calls the routine usrmat, which starts the corresponding element routine, depending on the element type. In the case of beam elements this is urmatb and for truss elements urmatt. Finally, the actual material routine is called, which the user can program himself. It is possible to have up to ten user materials defined in the subroutines umat41 to umat50. The user can implement arbitrary material models in these routines and, among other things, access the material parameters specified in the material card. In addition, the programmer may call further subroutines, which then return, for example, nodal coordinates or various element properties.

**Fig. 4:**
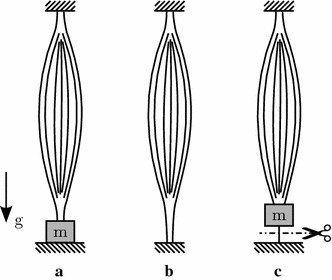
Illustration of the** a** concentric and** b** isometric contraction and the** c** quick release experiments

### Verification and validation set-up

For the Hill-type model parameters identification, a general test procedure requires three experimental set-ups: (a) concentric contraction, (b) isometric contraction and (c) quick release [12]. They are depicted in Fig. 4 and explained in detail below. A complete set of muscle parameters is almost never found in a single source since it is hard to perform all three tests in a row with the same muscle specimen, so a short literature survey is always required.

The validation conducted for the muscle model is based on mammalian muscle experiments. As there is no experimental data available for actual human muscle tissue, the model validation is based on piglet [16], cat [34] and rat [35] muscle experiments. The verification is done in comparison with an already existing implementation of the same muscle model in the Matlab based multi-body code Neweul-M^2^ [36]. Additionally, a comparison with the *MAT_156 muscle material from LS-DYNA is shown for the concentric contraction experiment. Data sets from all three experimental set-ups are available for the piglet muscle. For the other two species, a specific set-up for an isometric contraction experiment is applied, which was shown to be sufficient to determine all necessary Hill-type parameters for simulations [37]. An overview of all experimental set-ups is presented in Table 1. Furthermore all model parameters are given in tabular form for each validation case including references.

**Table 1:** Overview of all set-ups used for validation

	Piglet	Cat	Rat
Concentric contraction	X		
Isometric contraction	X	X	X
Quick release	X		

#### Concentric contraction experiment

In a concentric contraction, the muscle is shortened, which means that the distance between muscle origin and insertion point decreases, e.g. elbow flexion to lift a weight, see Fig. 4a. In the simulation set-up, the muscle element is orientated vertically and the upper node is fixed. Masses between $m= {0.1}$ kg and $m={1.8}$ kg are attached to the lower node in accordance with the experimental studies. At the beginning of the test, the muscle is relaxed and the mass is resting on a plane. Then the muscle is stimulated ($\text {STIM} = 1$) and starts to contract. At first no external motion is recorded as only the internal length $l_\text {CE}$ is decreasing. Once $F_\text {MTC}>F_\text {Gravity}$ the mass is lifted and the contraction velocity is recorded and compared to the experimental results from the piglet muscles.

#### Isometric contraction experiment

In an isometric contraction the muscle force is increased, while the length of the muscle is kept constant, see Fig. 4b. This contraction mode occurs, for example, when attempting to hold a heavy weight. In the simulation set-up both nodes of the muscle element are fixed. At the beginning of the test, there is no stimulation ($\text {STIM} = 0$), thus the muscle experiences only a minimal activity $q_0$. Starting from 0.1 s, the muscle is stimulated completely ($\text {STIM} = 1$) and relaxed again completely after 1.1 s ($\text {STIM} = 0$) in the piglet [16] and cat [34] experiments. In the rat experiments the muscle is only activated for a shorter time period of 300 ms [35]. In the piglet muscle experiments the isometric contraction was carried out for different fixed muscle lengths between 5.1 and 6.6 cm around the anatomical resting length of $l_\text {MTC,0} = l_\text {CE,opt} + l_\text {SEE,0}$. In the other experiments the isometric contraction was only tested for the anatomical resting length. In the results, the force vs. time curves are compared and also the differences resulting from the two activation dynamics implemented are analyzed.

#### Quick release experiment

The quick release is a combination of isometric and concentric contraction. In this set-up, the muscle is fixed at both ends at the beginning, it is then stimulated (STIM = 1) and isometric contraction occurs Fig. 4c. After 1 s the lower end of the muscle carrying a mass is released and is pulled up quickly due to the force built up during the isometric contraction. After a total time of 1.5 s the stimulation is switched off again (STIM = 0). As in the concentric case, the influence of the different masses is examined (*m* = 200, 400, 600, 800, 1000, and 1500 g) for the piglet muscles only [16].

## Simulation results

The verification and validation simulation results are shown in the following sections for piglet, rat and cat muscles. Using the piglet data, an additional comparison to the muscle model *MAT_156 already existing in LS-DYNA is shown and the differences resulting from the two distinctive muscle activation dynamics implemented are illustrated. To demonstrate the application of the model in AHBMs an example illustrating the routing capabilities is given using an elbow model extracted from the ViVA OpenHBM [3].

### Piglet calf muscle

For the piglets calf muscles results for concentric and isometric contraction and quick release experiments are available in [16]. As this is the most complete data set, it was also used for verification and a comparison with *MAT_156 and a comparison of the different activation dynamics available in the extended Hill-type muscle model. In Table 2 the parameters used for the piglet simulations are listed. The material card for LS-DYNA is found in Appendix "Material card for piglet simulations".

**Table 2:** Muscle parameters for the piglet simulations. See [16, Table 2, p. 68]

Activation dynamics (Zajac [11])	$q_0 [~]$ 1.0e−4	$\tau _\text {q} [s]$ 0.025	$\beta _\text {q} [~]$ 0.5			
Activation dynamics (Hatze [29])	$q_0 [~]$ 5.0e−3	*c*[ ] 1.373e−4	$\eta [~]$ 5.27e−4	*k*[ ] 2.9	*m*[ ] 11.3	
Isometric force	$F_\text {max} [N]$ 30.0	$l_\text {CE,opt} [m]$ 0.015	$\Delta W_\text {des} [~]$ 0.14	$\nu _\text {CE,des} [~]$ 3.0	$\Delta W_\text {asc} [~]$ 0.57	$\nu _\text {CE,asc} [~]$ 4.0
Force-velocity hyperbola	$A_\text {rel,0} [~]$ 0.1	$B_\text {rel,0} [1/s]$ 1.0	$S_\text {e} [~]$ 2.0	$F_\text {e} [~]$ 1.8		
PEE	$\mathcal {L}_\text {PEE,0} [~]$ 0.9	$\nu _\text {PEE} [~]$ 2.5	$\mathcal {F}_\text {PEE} [~]$ 1.0			
SEE	$l_\text {SEE,0} [m]$ 0.045	$\Delta U_\text {SEE,nll} [~]$ 0.1825	$\Delta U_\text {SEE,l} [~]$ 0.073	$\Delta F_\text {SEE,0} [N]$ 60.0		
SDE	$D_\text {SDE} [~]$ 0.3	$R_\text {SDE} [~]$ 0.01				

#### Concentric contraction

**Fig. 5:**
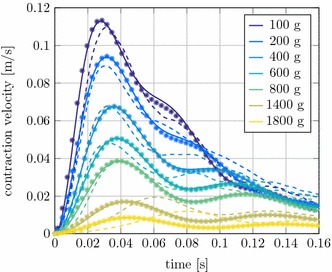
Concentric contraction velocity of the MTC of a piglet over time at different muscle loads.* Full line* LS-DYNA, *dots* Neweul-M^2^,* dashed line * experimental results from [16]

The numerical and experimental results are presented in Fig. 5. All curves are shifted in time so that the mass is pulled up or $F_\text {MTC}=F_\text {Gravity}$ occurs at $t=0~\text {s}$. As shown in the figure, the simulation results from LS-DYNA are very consistent with the experiments and the simulations with the muscle model in Neweul-M^2^. A comparison with the simulation data from [16] would give even better results. The differences between the simulation results from Neweul-M^2^ and LS-DYNA can presumably be attributed to different computational accuracies and integration methods for the differential equations. These simulations were both run with explicit integrators and a constant time step. Consequently, we can state that we have a correct muscle model implementation for the concentric contraction case.

**Fig. 6:**
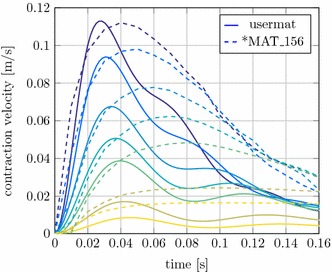
Comparison of the concentric contraction velocity between the extended Hill-type muscle model and *MAT_156.* Full line* extended Hill-type muscle model,* dashed line* *MAT_156. Colours are identical to Fig. 5

In addition a comparison with the muscle material model *MAT_156, already existing in LS-DYNA, is shown in Fig. 6. The initial contraction velocity provides similar results. Also, the maximum force value for low masses up to about 200 g is well approximated. The *MAT_156 material, however, shows significant weaknesses in speed decay and in the correct representation of the damping properties. At this point, it should be noted that further optimization of the material parameters might achieve better results. For these simulations the *MAT_156 parameters were derived from the previous work of [38]. This comparison shows the potential of the newly implemented muscle model and shows how it can help to deliver more realistic simulation results.

#### Isometric contraction

**Fig. 7:**
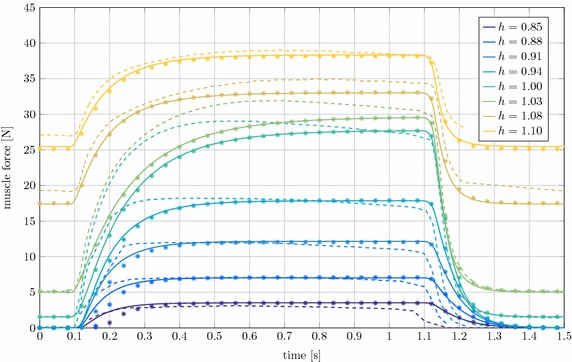
Force output of the MTC plotted versus time for different fixed stretch ratios *h* in isometric contraction.* Full line* LS-DYNA,* dots* Neweul-M^2^,* dashed line* experimental results

In Fig. 7 the numerical results for both the LS-DYNA and the Neweul-M^2^ simulations are depicted together with the experimental results. In the piglet experiments different starting length of the MTC $l_\text {MTC}$ were tested. In Fig. 7 the tests are differentiated by the stretch ratio $h = {l_\text {MTC}}/{l_\text {MTC,0}}$ of the starting length and the anatomical resting length $l_\text {MTC,0}$.

The comparison of the LS-DYNA results with the experimental data shows, that the muscle force for the inactive muscle in the time intervals ${t<0.1~\text {s}}$ and ${t>1.1~\text {s}}$ is underestimated if the muscle is lengthened considerably relative to the anatomical resting length ($h>1.05$). Also, a clear deviation in the force increase for the stretch ratios $h = 1.0$ and $h = 1.03$ exists, while the final force at $t= 1~\text {s}$ is met. Very similar differences are also present in the simulation results from [16]. According to this source, the proposed muscle model does not represent all internal dependencies of the Hill parameters correctly. Also potential history effects visible in the experimental curves, namely non-steady force plateaus, are made responsible for the differences. Furthermore, possible deficits in the identification of the parameters for the activation dynamics and the rise of $F_\text {CE}$ play a role. The comparison to Neweul-M^2^ shows high agreement with only slight deviations in the muscle activation interval for the ratios $h = 0.85, 0.88$ and 0.91. As it was the case in the concentric contraction, the differences in the results are larger for higher dynamics, which can probably be attributed to the different integration method for solving the differential equations.

The most important point illustrated by the isometric contraction in Fig. 7 is the strong dependence of the maximum isometric force on the muscular length. This finding is decisive for the application in AHBMs, since in this example deviations of approximately 15 mm in muscle length lead to differences in muscle force of more than 30 N.

#### Comparison of activation dynamics for isometric contraction

**Fig. 8:**
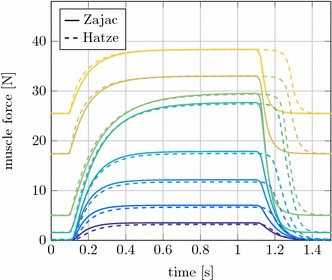
Comparison of muscle forces for Zajac and Hatze activation dynamics. Colours identical to Fig. 7

**Fig. 9:**
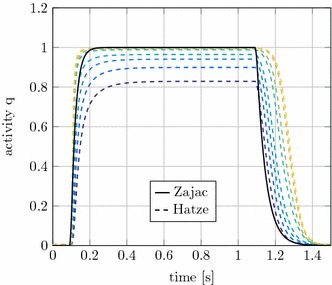
Comparison of the activation level *q* for Zajac and Hatze activation dynamics. Colours identical to Fig. 7

In the extended Hill-type muscle model two different approaches to describe the activation dynamics are implemented. In Figs. 8 and 9 these methods by Zajac [11] and Hatze [29] are compared in an isometric contraction.

The muscle force results with Hatze and Zajac activation differ mainly during muscle deactivation after $t=1.1~\text {s}$, see Fig. 8. The forces of muscles with a high stretch ratio decrease significantly later than the muscles that are shortened. The concentration of free $Ca^{2+}$ ions $\gamma _\text {rel}$ evolves similar to the activity level described by the differential equation of Zajac. $\gamma _\text {rel}$ increases slightly slower and takes about 0.1 s longer to decay. The larger influence is the dependence of the activation on the CE length through $\rho$ for the Hatze activation dynamics, see Fig. 9. By stretching the muscle, $l_\text {CE}$ is significantly longer for high h-ratios. As a result, $\rho$ will increase and the activity is rising faster for the stretched muscles. The stretch ratio and thus $\rho$ also affect the maximum activity level reached in the simulations. It can be seen in Fig. 9, that the maximum activity of the muscle with $h = 0.85$ is only about $82 \%$. For Zajac’s activation dynamics only one curve is found in Fig. 9, this is because Zajac’s activation dynamics is length-independent and therefore all activation curves are identical.

As the formulation of activation dynamics by Hatze is the more biofidelic and superior option [37], the comparison for rat and cat experiments will be done only with this dynamics.

#### Quick release

**Fig. 10:**
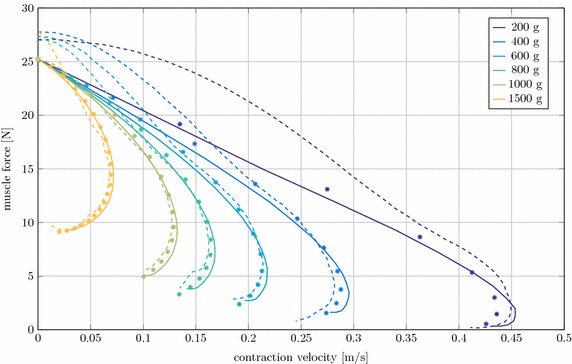
Force output of the MTC plotted versus contraction velocity at different muscle loads in quick release experiments.* Full line* LS-DYNA,* dots* Neweul-M^2^,* dashed line* experimental results

The quick release experiments are a combination of the two experiments above. Here the force produced by the MTC is analyzed versus the contraction velocity. In Fig. 10 the numerical results from LS-DYNA and Neweul-M^2^ as well as the experimental data is shown.

The isometric muscle force at zero velocity, i.e. before the muscle is released, is about 2 N lower than in the experiments. However, the results clearly approach the respective maximum contraction velocities. In [16] it is stated, that this is due to history effects within the tendon in the experiments that are not represented in the muscle model. The best agreement is achieved for a mass of 1000 g, where according to [16] the history effects were absent. The difference with Neweul-M^2^ is once again negligibly small for the bigger masses and slightly larger for the high velocities or smaller masses.

### Rat gastrocnemius medialis muscle

The experiments were done by Siebert et al [35] on the rat (Rattus norvegicus, Wistar) M. gastrocnemius medialis muscle. The parameters for the Hill-type muscle model are listed in [35, Table 4, p. 222] and the experimental set-up description is provided on page 218 of the same publication. Optimal Hatze activation dynamics parameters are listed in [37, Table 2, column 6, p. 278].

**Fig. 11:**
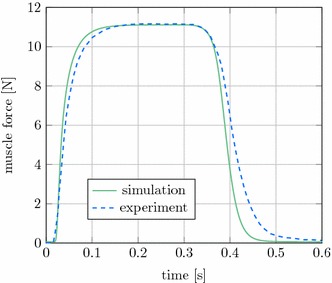
Isometric experimental and simulation results for a rat muscle

For convenience, they are collected in Table 3 and the LS-DYNA material card is given in Appendix "Material card for rat simulations". As seen in Fig. 11 the simulation results are in good agreement with the experimental results, being a little faster in the muscle deactivation slope.

**Table 3:** Muscle parameters for the rat simulations

Activation dynamics (Hatze [29])	$q_0 [~]$ 6.0e−3	*c*[ ] 1.373e−4	$\eta [~]$ 5.27e4	*k*[ ] 2.9	*m*[ ] 22.54	
Isometric force	$F_\text {max} [N]$ 11.2	$l_\text {CE,opt} [m]$ 0.0148	$\Delta W_\text {des} [~]$ 0.35	$\nu _\text {CE,des} [~]$ 1.5	$\Delta W_\text {asc} [~]$ 0.35	$\nu _\text {CE,asc} [~]$ 3.0
Force-velocity hyperbola	$A_\text {rel,0} [~]$ 0.06	$B_\text {rel,0} [1/s]$ 1.42	$S_\text {e} [~]$ 0.99	$F_\text {e} [~]$ 1.35		
PEE	$\mathcal {L}_\text {PEE,0} [~]$ 3.12	$\nu _\text {PEE} [~]$ 2.5	$\mathcal {F}_\text {PEE} [~]$ 2.0			
SEE	$l_\text {SEE,0} [m]$ 0.0123	$\Delta U_\text {SEE,nll} [~]$ 0.0425	$\Delta U_\text {SEE,l} [~]$ 0.017	$\Delta F_\text {SEE,0} [N]$ 4.48		
SDE	$D_\text {SDE} [~]$ 0.3	$R_\text {SDE} [~]$ 0.01				

### Cat soleus muscle

**Fig. 12:**
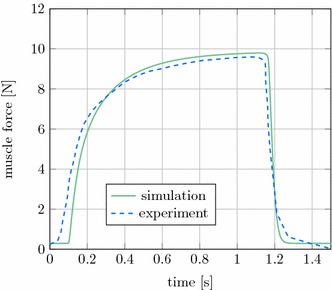
Isometric experimental and simulation results for a cat muscle

Mörl et al. [34] conducted the experiments on the cat soleus muscle. The parameters for the Hill-type muscle model are found in [34, Table 1, p. 5] with optimal Hatze activation dynamics parameters once more taken from [37]. They are also collected in Table 4 and a material card for LS-DYNA is provided in Appendix "Material card for cat simulations". The corresponding simulation results depicted in Fig. 12, are in excellent agreement with the experimental results, this time being a little faster in the muscle activation slope.

**Table 4:** Muscle parameters for the cat simulations

Activation dynamics (Hatze [29])	$q_0 [~]$ 1.0e−4	*c*[ ] 1.373e−4	$\eta [~]$ 5.27e4	*k*[ ] 2.9	*m*[ ] 22.54	
Isometric force	$F_\text {max} [N]$ 10.0	$l_\text {CE,opt} [m]$ 0.053	$\Delta W_\text {des} [~]$ 0.35	$\nu _\text {CE,des} [~]$ 1.5	$\Delta W_\text {asc} [~]$ 0.35	$\nu _\text {CE,asc} [~]$ 3.0
Force-velocity hyperbola	$A_\text {rel,0} [~]$ 0.07	$B_\text {rel,0} [1/s]$ 0.2	$S_\text {e} [~]$ 2.0	$F_\text {e} [~]$ 1.5		
PEE	$\mathcal {L}_\text {PEE,0} [~]$ 0.9	$\nu _\text {PEE} [~]$ 2.5	$\mathcal {F}_\text {PEE} [~]$ 2.0			
SEE	$l_\text {SEE,0} [m]$ 0.060	$\Delta U_\text {SEE,nll} [~]$ 0.0425	$\Delta U_\text {SEE,l} [~]$ 0.017	$\Delta F_\text {SEE,0} [N]$ 4.0		
SDE	$D_\text {SDE} [~]$ 0.3	$R_\text {SDE} [~]$ 0.01				

### Application in the ViVA OpenHBM Arm with routing

The extended Hill-type muscle model is applied in an arm model extracted from the ViVA OpenHBM [3]. The model includes bones, modelled as rigid bodies, and flexible flesh and skin of the upper extremity. We added the main flexors (biceps long and short heads, brachialis, brachioradialis, pronator teres and extensor carpi radialis) and extensors (triceps long, lateral and medial heads) of the elbow joint, which we idealized as a revolute joint. Here the via-point routing method is compared to simple direct line and lever approaches. Moreover a complete description of the set-up of the elbow model [39] and the choice of parameters for all muscles at the elbow [32] is out of scope for this publication.

The via-point method allows the selection of anatomical origin and insertion nodes for the muscles. As a result the modelled muscle length is almost identical to the anatomical muscle-tendon length. This enables the usage of anatomical data from literature for the muscle parameters. The routing is done in LS-DYNA using sliprings fixed to bones in certain positions in space. The routing parameters, i.e. the offset length of the muscle and the position of the via-point, can be chosen independently of the muscle parameters to match the anatomy. This approach makes it possible to model the muscle-tendon dynamics correctly and at the same time making the lever arm vs. joint angle curve and thus the resulting elbow torque more realistic.

**Fig. 13:**
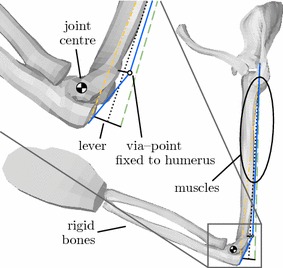
Different modeling strategies for the triceps at the elbow

**Fig. 14:**
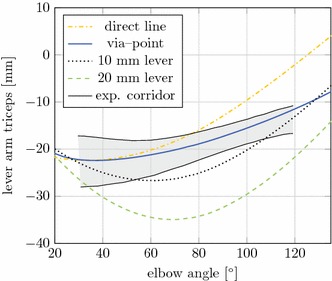
Lever arms for different modeling of the triceps. Colours are identical to Fig. 13. Corridor taken from [40]

In Fig. 13 different strategies for modeling the triceps are shown. A direct line of action approach, lever arms of 10 and 20 mm, and the via-point routing method are shown. In contrast to the other methods, the application of the via-point method can deliver correct lever arms for the complete range of motion of the elbow and fits the experimental corridor from [40] best, see Fig. 14. Additionally, the proposed via-point routing method improves the numerical stability of the model as it provides correct force application directions. Most importantly, the muscle dynamics is independent from the actual length of the combined elements (muscle + seatbelt, Fig. 2) and their path complexity in FE AHBMs. This is because of the separation of muscle and routing parameters in the FE model.

In comparison with the LS-DYNA *MAT_156 muscle model a 10 times speed-up is achieved for the validation simulations with one single muscle elements. As this set-up is clearly not very realistic, the ViVA arm simulations are repeated with *MAT_156 muscles to be comparable to the simulations with the extended Hill-type muscles. Here no speed-up is achieved, since most of the CPU time is used for the processing of the volumetric elements and the time needed to process truss elements is insignificant in comparison.

## Conclusion and outlook

The upcoming challenges in the field of automotive safety, namely active safety systems and autonomous driving, will require and benefit greatly from AHBMs. The Hill-type muscle model already existing in LS-DYNA has a limited accuracy because it lacks an internal degree of freedom and in addition is difficult to parameterize. Here, an extended Hill-type muscle model was implemented, verified and validated successfully. The source code, parameters and an example set-up for LS-DYNA are provided at https://zenodo.org/record/826209. The verification and validation was done in comparison with experimental data sets from piglet, cat and rat muscles. The results are in very good agreement with the experiments and the new muscle model improves the accuracy available for AHBMs in LS-DYNA considerably. Moreover, the muscle model incorporates the activation dynamics, essential for correct simulations on small time horizons of dynamic active movements. Additionally, a convenient option for routing the muscle around joints was proposed. By introducing an offset to the length of the muscle element, it is possible to route the muscle using e.g. the via-point method, while at the same time the muscle will display the correct dynamics of the full muscle. This also means, that the parameters for the muscle model can be set independently of the routing.

Although the current model allows to predict the gross dynamic contraction characteristics of biological muscles, it has its limitations. For one, it is a force element predicting a scalar force value which is then applied between origin and insertion, or redirected by via-points. Contact forces and resulting shifts in the force direction or their influence on the active muscle force [35] are neglected. Besides that, several physiological effects of the muscle contraction are currently not considered, starting with muscle-morphology specific parameters such as the pennation angle [41] or the fibre composition [42]. Also on the dynamic level, e.g., modelling the force-velocity relation for the eccentric (lengthening) contractions is difficult, as little data is available. Some of the data suggests more complex relations than modelled here for extensive strains [43], which, however, are not reached in our simulations. Furthermore, the experimentally found history effects causing force enhancement and force depression after stretch and shortening are currently not considered, but may be included in more extended approaches [44, 45]. Finally, the muscles model considers no mass or mass distribution, which, however, plays a role in dynamic contractions [46].

To utilize the full potential of the AHBMs, a control strategy for the activation of the muscles is needed. As a controller realization is not in the scope of this work, the authors recommend the review by [47] as a reliable source of information for muscle activations schemes and strategies in AHBMs. In principle, controllers are required which either maintain a desired position against perturbations or allow for the generation of a desired movement. Such controllers can be implemented in the current framework and may be easily added to the code provided in the Appendix.

With this, we provide a comprehensive and valid approach to implement an extended Hill-type muscle model in LS-DYNA, including muscle-tendon properties, biochemical activation dynamics, and muscle routing. By providing the code and the material cards, we hope that this will allow other researchers to work on more biofidelic AHBMs.

## Appendix A: LS-DYNA material cards for validation simulations

In the following sections, the material cards for the simulations of the piglet, cat and rat muscles are given in the SI unit system. The simulations were set up with single muscle elements with a length of $l_\text {MTC} = l_\text {CE,opt} + l_\text {SEE0}$ where applicable. The parameters are taken from [14, 16, 25, 29, 34, 35, 37]. LS-DYNA uses the density, bulk modulus and shear modulus to determine the time-step for the simulation. The density was set to $1\times 10^{-6}$ kg $\mathrm{m^{-3}}$ and the bulk modulus and shear modulus were adjusted to achieve a time-step smaller than $1\times 10^{-4}$ s.

## Appendix B: Material cards description and corresponding symbols

The material cards for the user-defined material are described in detail below. The first two cards are pre-defined by LS-DYNA. In the cards 3–6 the parameters for the implemented muscle model are defined. They are listed together with the corresponding symbols used in the respective paper. Parameters marked with $^*$ are affecting the automatic calculation of the time-step by LS-DYNA. Parameters marked with $\dagger$ are affected by changes in the unit system.

**Table:**

Card 1	1	2	3	4	5	6	7	8
Variable	MID	RO	MT	LMC	NHV	IORTH	IBULK	IG
Default	–	1E-6	41	32	15	0	31	32

**Table:**

Variable	Description
MID	Material identification. A unique number or label not exceeding eight characters must be specified
RO$^*\dagger$	Mass density. Not used by the material model
MT	User material type. In this case 41 must be defined
LMC	Length of material constant array. For this material 32 must be set
NHV	Number of history variables to be stored. 15 are required for this material
IORTH	EQ.1: if the material is orthotropic
	EQ.2: if material is used with spot weld thinning
	EQ.3: if material is orthotropic and used with spot weld thinning
IBULK	Adress of bulk modulus in material constants array
IG	Adress of shear modulus in material constants array

**Table:**

Card 2	1	2	3	4	5	6	7	8
Variable	IVECT	IFAIL	ITHERM	IHYPER	IEOS	LMCA		
Default	0	1	-	–	–	–	–

**Table:**

Variable	Description
IVECT	Vectorization flag (on = 1). A vectorized user subroutine must be supplied
IFAIL	Failure flag
	EQ.0: No failure
	EQ.1: Allows failure of shell and solid elements
	LT.0: |IFAIL| is the address of NUMINT in the material constants array
ITHERM	Temperature flag (on = 1). Compute element temperature
IHYPER	Deformation gradient flag (on = 1 or −1, or 3). Compute deformation gradient, see LSTC Appendix A: LS-DYNA material cards for validation simulations
IEOS	Equation of state (on = 1)
LMCA	Length of additional material constant array

**Table:**

Card 3	1	2	3	4	5	6	7	8
Variable	Act	STIM	q0	tauq/c	bq/eta	k	m	lOffset
Default	–	–	–	–	–	–	–	0

**Table:**

**Variable**	**Symbol**	**Description**
Act		EQ.0.0: Input of activation values
		EQ.1.0: Calculation of activation with Zajac depending on stimulation
		EQ.2.0: Calculation of activation with Hatze depending on stimulation
STIM	*STIM*	LT.0.0: Constant stimulation or activation level. Depending on Act
		GT.0.0: LCID specifing the stimulation or activation
q0	${q_{0}}$	Minimum value of activation *q*
tauq $\dagger$ / c	$\tau _\text {q}$ / c	If ACT.EQ.1.0: time constant of rising activation
		If ACT.EQ.2.0: Hatze constant *c*
bq / eta	$\beta _\text {q}$/ $\eta$	If ACT.EQ.1.0: ratio between $\tau _\text {q}$ and time constant of falling activation
		If ACT.EQ.2.0: Hatze constant $\eta$
k	*k*	If ACT.EQ.2.0: Hatze constant *k*
m	*m*	If ACT.EQ.2.0: Hatze constant *m*
lOffset $\dagger$	$l_\text {Offset}$	Muscle length offset added to beam length before calculation of the muscle. $l_{\text {MTC},i} = l_{\text {Beam},i} + l_\text {Offset}$

**Table:**

Card 4	1	2	3	4	5	6	7	8
Variable	Fmax	lCEopt	dWdes	nCEd	dWasc	nCEa	Arel0	Brel0
Default	–	–	–	–	–	–	–	–

**Table:**

**Variable**	**Symbol**	**Description**
Fmax $\dagger$	$F_\text {max}$	Maximum isometric force
lCEopt $\dagger$	$l_\text {CE,opt}$	Optimal fibre length
dWdes	$\Delta W_\text {des}$	Width of $F_\text {isom}(l_\text {CE})$ on descending limb
nCEd	$\nu _\text {CE,des}$	Exponent of $F_\text {isom}(l_\text {CE})$ on descending limb
dWasc	$\Delta W_\text {asc}$	Width of $F_\text {isom}(l_\text {CE})$ on ascending limb
nCEa	$\nu _\text {CE,asc}$	Exponent of $F_\text {isom}(l_\text {CE})$ on ascending limb
Arel0	$A_\text {rel,0}$	Maximum value of $A_\text {rel}$
Brel0 $\dagger$	$B_\text {rel,0}$	Maximum value of $B_\text {rel}$

**Table:**

Card 5	1	2	3	4	5	6	7	8
Variable	Secc	Fecc	LPEE0	nPEE	FPEE	lSEE0	dUSnll	dUSl
Default	–	–	–	–	–	–	–	–

**Table:**

Variable	Symbol	Description
Secc	$S_\text {e}$	Step in inclination of $F_\text {CE}(\dot{l}_\text {CE}=0)$ between eccentric and concentric force-velocity relations
Fecc	$F_\text {e}$	Coordinate of pole in $l_\text {CE}(F_\text {CE})$ normalised to $F_\text {max}qF_\text {isom}(l_\text {CE})$ for $l_\text {CE} > 0$
LPEE0	$\mathcal {L}_\text {PEE,0}$	Rest length of PEE normalised to $l_\text {CE,opt}$
nPEE	$\nu _\text {PEE}$	Exponent of $F_\text {PEE}(l_\text {CE})$
FPEE	$\mathcal {F}_\text {PEE}$	Force of PEE if $l_\text {CE}$ is stretched to $\Delta W_\text {des}$
lSEE0 $\dagger$	$l_\text {SEE,0}$	Rest length of SEE
USnll	$\Delta U_\text {SEE,nll}$	Relative stretch at non-linear-linear transition in $F_\text {SEE}(l_\text {SEE})$
duSl	$\Delta U_\text {SEE,l}$	Relative stretch in linear part for force increase $\Delta F_\text {SEE,0}$

**Table:**

Card 6	1	2	3	4	5	6	7	8
Variable	dFSEE0	Damp	Damp1	Damp2	Output	dtOut	IBULK$^*$	IG$^*$
Default	-	3	-	-	0	0	-	-

**Table:**

Variable	Symbol	Description
dFSEE0 $\dagger$	$\Delta F_\text {SEE,0}$	Force at non-linear-linear transition in $F_\text {SEE}(l_\text {SEE})$
Damp		EQ.1.0: parallel damping
		EQ.2.0: Serial damping
		EQ.3.0: Serial force dependent damping
		Else: No damping
Damp1	$d_\text {PE} \dagger$	If Damp.EQ.1.0: damping coefficient of PE
	$d_\text {SE} \dagger$	If Damp.EQ.2.0: damping coefficient of SE
	$D_\text {SDE}$	If Damp.EQ.3.0: dimensionless factor to scale $d_\text {SE,max}$
Damp2	$R_\text {SDE}$	If Damp.EQ.3.0: minimum value of $d_\text {SE}$ normalised to $d_\text {SE,max}$
Output		Definition of desired output content of outputfile *fort.(idele)* for each muscle element:
		EQ.0. no outputfile
		EQ.1. basic output (idele, tt, ncycle, q)
		EQ.2. basic output and force data
		($$idele...,F_\text {MTC}, F_\text {SEE}, F_\text {SDE}, F_\text {CE}, F_\text {PEE}, F_\text {isom}$$)
		EQ.-1. basic output and length data
		$$(idele..., l_\text {MTC}, l_\text {CE}, \dot{l}_\text {MTC}, \dot{l}_\text {CE})$$
		EQ.-2. basic output and force and length data
		$$(idele ..., F_\text {MTC} ..., l_\text {MTC} ...)$$
dtout $\dagger$		Timestep of outputile *fort.(idele)*
IBULK$^*$		Bulk modulus. Needed by LS-DYNA to calculate time-step automatically
IG$^*$		Shear modulus. Needed by LS-DYNA to calculate time-step automatically
